# An overview of gene regulation in bacteria by small RNAs derived from mRNA 3′ ends

**DOI:** 10.1093/femsre/fuac017

**Published:** 2022-04-07

**Authors:** Falk Ponath, Jens Hör, Jörg Vogel

**Affiliations:** Helmholtz Institute for RNA-based Infection Research (HIRI), Helmholtz Centre for Infection Research (HZI), D-97080 Würzburg, Germany; Institute for Molecular Infection Biology, University of Würzburg, D-97080 Würzburg, Germany; Helmholtz Institute for RNA-based Infection Research (HIRI), Helmholtz Centre for Infection Research (HZI), D-97080 Würzburg, Germany; Institute for Molecular Infection Biology, University of Würzburg, D-97080 Würzburg, Germany

**Keywords:** 3′ UTR, sRNA, post-transcriptional control, regulatory networks, bacteria

## Abstract

Over the past two decades, small noncoding RNAs (sRNAs) that regulate mRNAs by short base pairing have gone from a curiosity to a major class of post-transcriptional regulators in bacteria. They are integral to many stress responses and regulatory circuits, affecting almost all aspects of bacterial life. Following pioneering sRNA searches in the early 2000s, the field quickly focused on conserved sRNA genes in the intergenic regions of bacterial chromosomes. Yet, it soon emerged that there might be another rich source of bacterial sRNAs—processed 3′ end fragments of mRNAs. Several such 3′ end-derived sRNAs have now been characterized, often revealing unexpected, conserved functions in diverse cellular processes. Here, we review our current knowledge of these 3′ end-derived sRNAs—their biogenesis through ribonucleases, their molecular mechanisms, their interactions with RNA-binding proteins such as Hfq or ProQ and their functional scope, which ranges from acting as specialized regulators of single metabolic genes to constituting entire noncoding arms in global stress responses. Recent global RNA interactome studies suggest that the importance of functional 3′ end-derived sRNAs has been vastly underestimated and that this type of cross-regulation between genes at the mRNA level is more pervasive in bacteria than currently appreciated.

## Teaser

There are a growing number of examples of bacterial mRNAs that produce small RNAs cleaved from their own 3′ ends to post-transcriptionally control other genes.

## Introduction

Bacteria regulate and fine-tune gene expression at all levels, including post-transcriptional control mechanisms acting at the mRNA level. Evidence for post-transcriptional control of bacterial genes dates back to the early days of molecular biology (Wagner and Simons 1994). However, unlike gene control at the DNA level, which one readily associates with transcription factors (TFs), an abundant class of molecular factors that selectively target mRNAs was long unknown. This began to change when systematic searches in the early 2000s discovered hitherto unexpected numbers of small regulatory RNAs (sRNAs) in *Escherichia coli* (Argaman *et al*. 2001,Rivas *et al*. 2001, Wassarman *et al*. 2001, Chen *et al*. 2002 , Vogel *et al*. 2003 ).

The design of these foundational screens has strongly influenced our view of sRNAs. Based on the few sRNAs known at that time (Wassarman *et al*. 1999), these screens made two general assumption: (i) that sRNAs were primary transcripts of 100–200 nucleotides in length, produced from independent noncoding genes with their own promoter and Rho-independent terminator; and (ii) that they were encoded in otherwise empty intergenic regions (IGRs), reasonably spaced from the next open reading frame (ORF). Sequence conservation in the few other available enterobacterial genomes was considered an additional hallmark of a true sRNA gene (Argaman *et al*. 2001, Rivas *et al*. 2001, Wassarman *et al*. 2001).

Numerous sRNAs from these screens have since been functionally characterized in both *E. coli* and *Salmonella enterica*, and assigned a cellular pathway, molecular mechanism and physiological function (Hör *et al*. 2020a). Mechanistically, almost every one of them has turned out to regulate multiple mRNAs by short, imperfect base pairing. Target recognition typically involves 8–10 strongly conserved bases in the sRNA, its so-called ‘seed region’ (Storz *et al*. 2011). The major mode of action of sRNAs is repression of protein synthesis through hindering access to an mRNA's ribosome binding site (RBS), which additionally often leads to degradation of the mRNA. Importantly, most well-characterized sRNAs also require an RNA-binding protein (RBP) such as Hfq or ProQ for both intracellular stability and mRNA targeting (Holmqvist and Vogel 2018)—a fact that can be utilized for the detection of new sRNAs, as discussed later.

The overall picture emerging from these studies is that among the ∼300 sRNAs annotated in *E. coli* and *Salmonella*, there is a conserved set of 20–30 sRNA genes with core functions (Hör *et al*. 2020a). Similar to core TFs acting on the promoters of multiple genes within conserved regulons, these core sRNAs often target large suites of mRNAs with related functions. Importantly, sRNAs often substantially expand the regulatory scope of the TFs they are regulated by. This is especially apparent in the case of sigma factors, which are intrinsically restricted to activating genes; by activating an sRNA, a sigma factor can then also repress gene expression, albeit indirectly, at the post-transcriptional level. As such, sRNAs endow these regulons with a ‘noncoding arm’ that complements the TF-driven ‘coding arm’ (Gogol *et al*. 2011).

Two decades after the initial screens, sRNA genes are now known to provide a noncoding arm in many major stress regulons and signaling pathways. In γ-proteobacteria, for example, sRNA functions run the gamut from control of iron availability, cell surface or envelope stress, and sugar fluctuations, to the regulation of virulence gene expression in response to population density (Storz and Papenfort 2019). However, the more evident the central role of these sRNAs became, the more puzzling it was that there were several fundamental pathways with no obvious associated sRNA. For example, the Cpx response to inner membrane stress was known to rely on Hfq, indicating the involvement of an sRNA, but there was no intergenic sRNA gene with a conserved binding site for the major response regulator, CpxR (Vogt *et al*. 2014, Chao and Vogel 2016). Could it be that there was another source of sRNAs?

As outlined in this review and related previous ones (Miyakoshi *et al*. 2015b ,Adams and Storz 2020), we now know that fragments derived from 3′ untranslated regions (UTRs) of bacterial mRNAs provide another abundant class of sRNAs whose full scope and importance we are only beginning to understand. These 3′ UTR-derived sRNAs constitute a previously unknown layer of gene control in which mRNAs influence each other's expression without changes at the level of transcription. We will briefly summarize how these sRNAs were discovered, how they are produced in the cell, highlight several well-characterized examples and discuss emerging principles in the functional relationship with their parental mRNAs before ending with an outlook on where this exciting area of bacterial RNA biology may lead us in the next few years.

## Small RNAs from mRNA regions: a bit of history

**Figure 1.**
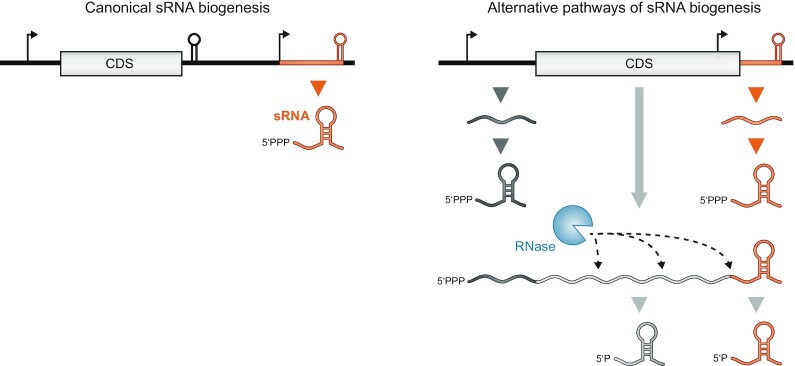
The many different sources of sRNAs as ‘parallel transcription output’. The canonical sRNA biogenesis pathways (left column) refer to sRNA production by transcription of stand-alone noncoding genes (right) located next to protein-coding genes (left). Alternative pathways (right column) produce sRNAs from mRNA loci by premature transcription termination in the 5′ region, by transcription starting inside the coding sequence (CDS) but using the same terminator or by mRNA processing. The focus of this review is on sRNAs that accumulate as 3′ end processing products of mRNAs and thus carry a monophosphate at their 5′ end.

Hints that noncoding genes may not be the sole origin of sRNAs were provided by some of the first studies that focused on IGRs. Performed in *E. coli*, these experimental screens recovered unusually abundant, defined small fragments derived not only from rRNAs and tRNAs, but also from different regions of mRNAs (Wassarman *et al*. 2001, Vogel *et al*. 2003, Kawano *et al*. 2005). 3′ UTR-derived sRNAs, in particular, showed expression patterns that differed from their corresponding mRNAs, suggesting independent functions (Kawano *et al*. 2005). Their intracellular half-lives were similar to those of known regulatory sRNAs (Vogel *et al*. 2003). Some of these fragments, such as SroC, were even conserved in the few other enterobacterial genomes available at the time. Although somewhat speculative, these observations were conceptualized as ‘parallel transcriptional output’ (Vogel *et al*. 2003) whereby the same transcriptional unit provides both a coding (mRNA) and a noncoding (sRNA) function (Fig. 1). However, the only promising candidate at the time was the long-known prophage-derived DicF sRNA, which, when overexpressed, blocks FtsZ protein synthesis leading to cell filamentation (Faubladier *et al*. 1990, Tétart and Bouché 1992).

The subsequent years yielded more evidence for functional sRNAs originating from protein-coding genes. Work in *Listeria monocytogenes* showed that sRNAs from mRNA 5′ regions could repress the synthesis of virulence regulator PrfA *in trans* (Loh *et al*. 2012). These sRNAs are derived from 5′ UTR-borne S-adenosyl methionine riboswitches (Loh *et al*. 2009), echoing earlier observations in *E. coli*, where flavin and thiamine riboswitches were found to produce stable 5′ mRNA fragments (Vogel *et al*. 2003). Concerning the 3′ end, work in *Vibrio cholerae* described the MicX sRNA, which is transcribed from an ORF-internal promoter and accumulates as a stable ∼190-nt processed species that roughly corresponds to the 3′ UTR of the ORF (Davis and Waldor 2007).

With the introduction of RNA-seq, comprehensive profiling of cellular ligands of Hfq by a RIP-seq approach revealed mRNA 3′ ends as a large potential sRNA pool (Sittka *et al*. 2008, Chao *et al*. 2012). In addition to determining the exact boundaries of enriched transcripts for the first time, RNA-seq also revealed their relative abundance on Hfq. Indeed, stable 3′ UTR fragments were found to occupy a substantial fraction of cellular Hfq (Chao *et al*. 2012). By that time, it had become clear that Hfq in the cell was present in a limiting concentration, resulting in competition amongst RNAs for access to this central sRNA chaperone (Fender *et al*. 2010). Thus, these abundant Hfq-associated 3′ UTR fragments were likely to have a cellular function. Moreover, the Rho-independent terminator structure at the 3′ end of sRNAs had been shown to be important for Hfq binding (Zhang *et al*. 2003, Sauer and Weichenrieder^2011^, Morita *et al*. 2017). Obviously, such structure was also present at the 3′ end of many mRNAs. Together, this suggested a scenario in which final products of mRNA turnover or processing accumulated on Hfq to exert an independent function as sRNAs (Chao *et al*. 2012).

Yet, not every Hfq-enriched 3′ UTR was a product of mRNA processing. There were several cases like the aforementioned MicX where sRNA transcription starts within the upstream CDS. For example, *Salmonella* DapZ, which acts to regulate amino acid synthesis and transport genes, possesses a conserved promoter that lies just upstream of the stop codon of the protein-coding gene *dapB* (Chao *et al*. 2012). Similarly, the 80-nt MicL sRNA, a conserved σ^E^-dependent repressor of Lpp synthesis in *E. coli*, is processed from a ∼300-nt precursor whose transcription starts in the middle of *cutC* (Guo *et al*. 2014, Updegrove *et al*. 2019). In the Gram-positive bacterium *Lactococcus lactis*, the ∼66-nt ArgX sRNA is transcribed from the 3′ end of *argR* and downregulates the *arc* operon involved in arginine metabolism (van der Meulen *et al*. 2019). For a very recent example, the cyanobacterium *Synechocystis* sp. PCC 6803 was found to express an sRNA called ApcZ from the 3′ end of a key operon involved in the collection of light energy, which acts to regulate the expression of a protein involved in energy dissipation (Zhan *et al*. 2021). Such sRNAs with ORF-embedded promoters will still accumulate when transcription of the overlapping protein-coding gene is inactivated, and unless they undergo further processing as do the MicL or MicX sRNAs (Davis and Waldor 2007, Guo *et al*. 2014), they will carry the characteristic 5′ triphosphate group of primary transcripts.

The Hfq ligand maps left plenty of strong sRNA candidates that were obviously cleaved from longer mRNAs, and their characterization in *Salmonella* and *E. coli* soon revealed Hfq-dependent functions as *trans*-acting regulators of other transcripts, both mRNAs and sRNAs (Miyakoshi *et al*. 2015a, Chao and Vogel 2016, Grabowicz *et al*. 2016). At the same time, work in *Streptomyces coelicolor* revealed Hfq-independent, 3′ UTR-mediated repression for two superoxide dismutase mRNAs (Kim *et al*. 2014). In addition, the newly discovered global RNA-binding properties of ProQ provided yet another set of potentially functional 3′ mRNA fragments (Smirnov *et al*. 2016, Holmqvist *et al*. 2018, Melamed *et al*. 2020). Together, these observations led to the notion that mRNA crosstalk, increasingly investigated in eukaryotes (Tay *et al*. 2014), might be quite common in bacteria, too. To date, about a dozen sRNAs from mRNA 3′ ends have been functionally characterized in phylogenetically diverse bacteria (Table 1).

**Table 1.** An overview of discussed 3′ UTR-derived sRNAs.

sRNA	Parental mRNA	Main ribonuclease(s) involved in biogenesis	Target(s)	Organism(s)	References
CpxQ	*cpxP*	RNase E	*nhaB*, *skp*, *agp*, *ydjN*, *fimA*	Enterobacteriales	(Bianco *et al*. 2019, Chao and Vogel 2016, Grabowicz *et al*. 2016, Melamed *et al*. 2016)
DicF	*ydfABC-dicF-dicB-ydfD*	RNase E & RNase III	*pchA* *ftsZ*, *manX*	EHEC *E. coli* K12	(Azam and Vanderpool 2018, Balasubramanian *et al*. 2016, Bouché and Bouché 1989, Faubladier *et al*. 1990, Melson and Kendall 2019, Murashko and Lin-Chao 2017, Tétart and Bouché 1992)
SdhX	*sdhCDAB-sucABCD*	RNase E	*ackA* *fumB*, *yfbV* *fdoG*, *katG*	*Salmonella*/*E. coli* K12 *Salmonella* *E. coli* K12	(Cronan and Laporte 2005, De Mets *et al*. 2019, Melamed *et al*. 2016, 2020, Miyakoshi *et al*. 2019, Zhang *et al*. 2003)
SroC	*gltIJKL*	RNase E	*fliE*, *GcvB*	*Salmonella*	(Fuentes *et al*. 2015, Miyakoshi *et al*.2015a ; Vogel *et al*. 2003)
NarS	*narK*	RNase E	*nirC*	*Salmonella*	(Wang *et al*. 2020)
MalH	*malEFG*	RNase E	*ompC*, *ompA*	Enterobacteriales	(Iosub *et al*. 2021)
FarS	*fabB*	RNase E	*fadE*	*Vibrio cholerae*	(Huber *et al*. 2020)
OppZ	*oppABCDF*	RNase E	*oppBCDF*	*Salmonella*	(Hoyos *et al*. 2020)
RaiZ	*raiA*	RNase E	*hupA*	*Salmonella*	(Chao *et al*. 2012, Holmqvist *et al*. 2018, Melamed *et al*. 2020, Smirnov *et al*. 2016, 2017b, Westermann *et al*. 2019)
s-SodF	*sodF*	Unknown; not RNase E or RNase III	*sodN*	*Streptomyces coelicolor*	(Kim *et al*. 2014)
RsaC	*mntABC*	RNase III	*sodA*	*Staphylococcus aureus*	(Lalaouna *et al*. 2019)
RsaG	*uhpT*	RNase J1/J2	*rex*, *ldh1*, RsaI	*Staphylococcus aureus*	(Desgranges *et al*. 2021)
SorX	*RSP_0847*	Unknown	*potA*	*Rhodobacter sphaeroides*	(Peng *et al*. 2016)
PcrX	*pufQBALMX*	RNase E	*pufLMX*	*Rhodobacter sphaeroides*	(Eisenhardt *et al*. 2018)

## Biogenesis

There is currently no evidence for specialized biogenesis factors for 3′ UTR-derived sRNAs, and they often appear to constitute a terminal fragment of normal mRNA decay. A global analysis of cellular RNA 5′ ends before and after inactivation of RNase E (so-called TIER-seq) in *Salmonella* indicated that most 3′ UTR-derived sRNAs are generated by this major endoribonuclease (Chao *et al*. 2017). TIER-seq analyses in the model proteobacteria *V. cholerae* and *Rhodobacter sphaeroides* came to similar conclusions (Förstner *et al*. 2018, Hoyos *et al*. 2020). Despite being an apparent product of typical RNA decay, the cleavage generating the final 5′ end tends to be well defined, and often occurs near the stop codon of the upstream ORF.

**Figure 2.**
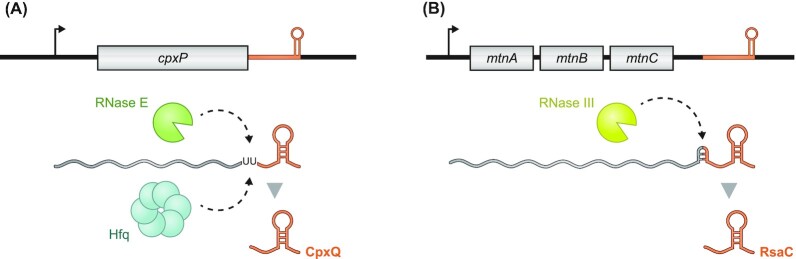
Biogenesis of 3′ UTR-derived sRNAs in Gram-negative and -positive bacteria. **(A)** In Gram-negative bacteria, the major endonuclease RNase E is the primary nuclease to produce 3′ UTR-derived sRNAs from their parental mRNAs, as shown here for the CpxQ sRNA and the *cpxP* mRNA. In this case, the Hfq protein is also required for biogenesis. **(B)** While RNase E is lacking in Gram-positive bacteria, RNase III was shown to free the RsaC sRNA from the *mntABC* operon mRNA by recognizing a double-stranded RNA structure.

RNase E-mediated biogenesis has been particularly well studied for the Hfq-dependent CpxQ sRNA, which—as described in the next section—is generated from the mRNA of the stress response protein CpxP. Mutation of an internal conserved RNase E cleavage site near the 3′ end of the *cpxP* mRNA (Fig. 2A) abrogates CpxQ production *in vivo* . Importantly this has little effect on the parental *cpxP* mRNA (Chao and Vogel 2016). *In vitro* reconstitution experiments suggested that RNase E and Hfq are the two key factors for generating CpxQ from the processed 5′P-*cpxP* mRNA. Hfq may have a dual role in this process: it enhances the precision of the required RNase E cleavage while it also protects the final CpxQ species from further degradation (Chao and Vogel 2016).

Gram-positive bacteria have a very different set of RNases than Gram-negative bacteria and do not encode RNase E (Durand and Condon 2018, Bechhofer and Deutscher 2019). Biogenesis of 3′ UTR-derived sRNAs must therefore occur via a different mechanism, as in the case of the *Staphylococcus aureus* RsaC sRNA that was shown to be generated by the double-strand specific endoribonuclease RNase III (Lioliou *et al*. 2012) (Fig. 2B). It has been argued in a recent review (Mediati *et al*. 2021) that the smaller number of processed 3′ UTR-derived sRNAs in Gram-positive bacteria might be due to the presence of 5′ → 3′ exoribonucleolytic activity. Specifically, Gram-positive bacteria possess RNase J1, which fully degrades mRNAs from the 5′ end in one go, resulting in fewer stable intermediates for evolution to act on and limiting the development of 3′ UTR-derived sRNAs. Nonetheless, there is a recent report in a Gram-positive bacterium of how blockage of 5′ → 3′ exoribonucleolytic activity by a stable hairpin structure in the 3′ region of a longer polycistronic mRNA generates an sRNA; this is an interesting analogy to the actions of RNase E in Gram-negative bacteria (Desgranges *et al*. 2021).

In the following, we will highlight well-studied examples of sRNAs that are generated by mRNA 3′ end processing and describe their function (Fig. 1). Moreover, recently developed global RNA interactome methods will be discussed to argue that there is still much to learn about mRNA crosstalk mediated by 3′ UTR-derived sRNAs.

## CpxQ: the noncoding arm of the inner membrane stress response

**Figure 3.**
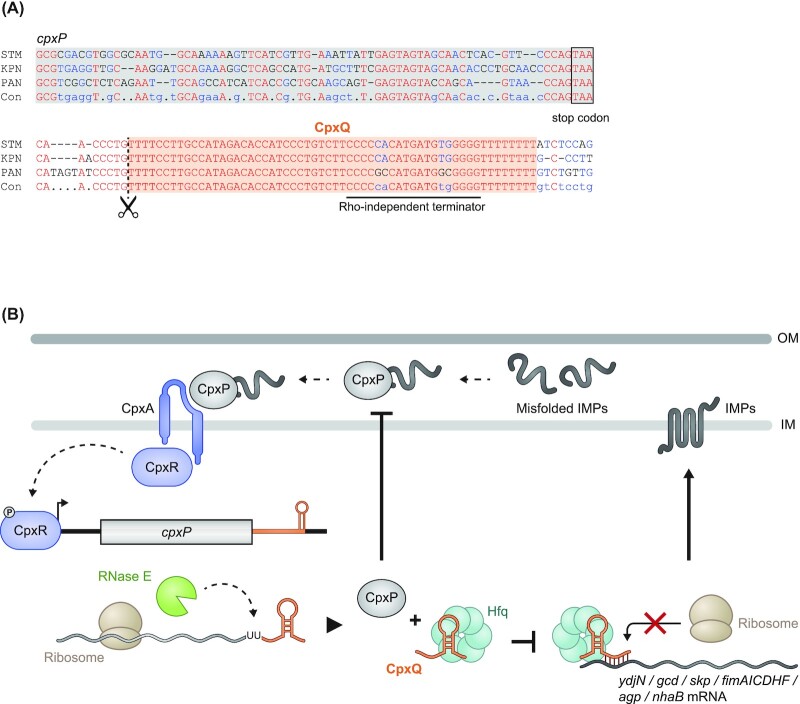
CxpQ as a well-understood example of 3′ UTR-derived sRNAs. **(A)** Nucleotide sequence alignment highlighting the strong conservation of the CpxQ sRNA in comparison to the 3′ region of the *cpxP* mRNA (STM: *S*. Typhimurium; KPN: *Klebsiella pneumoniae*; PAN: *Pantoea* spp.; Con: consensus sequence). **(B)** Inner membrane (IM) stress leads to the phosphorylation of the transcription factor CpxR. Phosphorylated CpxR activates transcription of *cpxP* mRNA that is translated, yielding CpxP protein, and processed by RNase E to yield CpxQ sRNA. While CpxP is involved in the degradation of misfolded inner membrane proteins (IMPs), CpxQ acts as a post-transcriptional regulator by repressing the translation of several IMPs. Thus, both the coding and noncoding parts of the *cpxP* mRNA act cooperatively to maintain IM homeostasis in the Cpx pathway.

The ∼60-nt CpxQ sRNA is a poster child for 3′ UTR-derived sRNAs as its discovery exemplifies the importance of both looking beyond IGRs for a missing sRNA in a conserved stress response and paying attention to unusually high sequence conservation at the 3′ end of bacterial genes. As previously mentioned, CpxQ is cleaved off the mRNA of CpxP, a protein with an important role in the stress response to misfolded inner membrane proteins (IMPs) (Chao and Vogel 2016, Grabowicz *et al*. 2016). CpxQ spans almost the entire *cpxP* 3′ UTR. Not only is this region exceptionally conserved amongst enterobacteria (Fig. 3A), it is also more conserved than any other region of the *cpxP* gene. As to function, CpxQ acts by Hfq-dependent base pairing to repress the synthesis of several IMPs including the NhaB Na^+^/H^+^ antiporter, thus limiting the loss of membrane potential under stress (Chao and Vogel 2016).

CpxQ solved a conundrum. It had been known that *hfq* mutants experience chronic stress at both the outer membrane (OM) and the inner membrane (IM), strongly suggesting that Hfq-dependent sRNAs were involved in maintaining homeostasis at both of these two membranes. However, while sRNAs had been found for the OM-related σ^E^ response (Johansen *et al*. 2006, Papenfort *et al*. 2006, Thompson *et al*. 2007), sRNA genes linked to the IM-related Cpx response remained unknown. The discovery of CpxQ explained this puzzling observation, showing that an mRNA 3′ fragment served as the elusive noncoding arm in the Cpx response to IM stress. Thus, both of these two major stress pathways employ Hfq-dependent sRNAs, yet of different nature, to counteract problematic overproduction of membrane proteins (Fig. 3B).

Interestingly, CpxQ also cross-connects these stress responses at the level of the σ^E^-induced Skp protein, a periplasmic chaperone that binds unfolded OMPs. Skp is known to accidently mistarget OMPs into the IM, causing membrane depolarization (Grabowicz *et al*. 2016). CpxQ counteracts this potential toxicity by downregulating Skp production (Chao and Vogel 2016, Grabowicz *et al*. 2016). Recent studies have further expanded the targetome of CpxQ, showing that this sRNA also represses the *cfa* mRNA, which encodes cyclopropane fatty acid synthetase (Melamed et *al*. 2016, Bianco *et al*. 2019). If and how this regulation expands the function of CpxQ in protecting IM integrity needs to be explored further.

## DicF: more than just a prophage function

**Figure 4.**
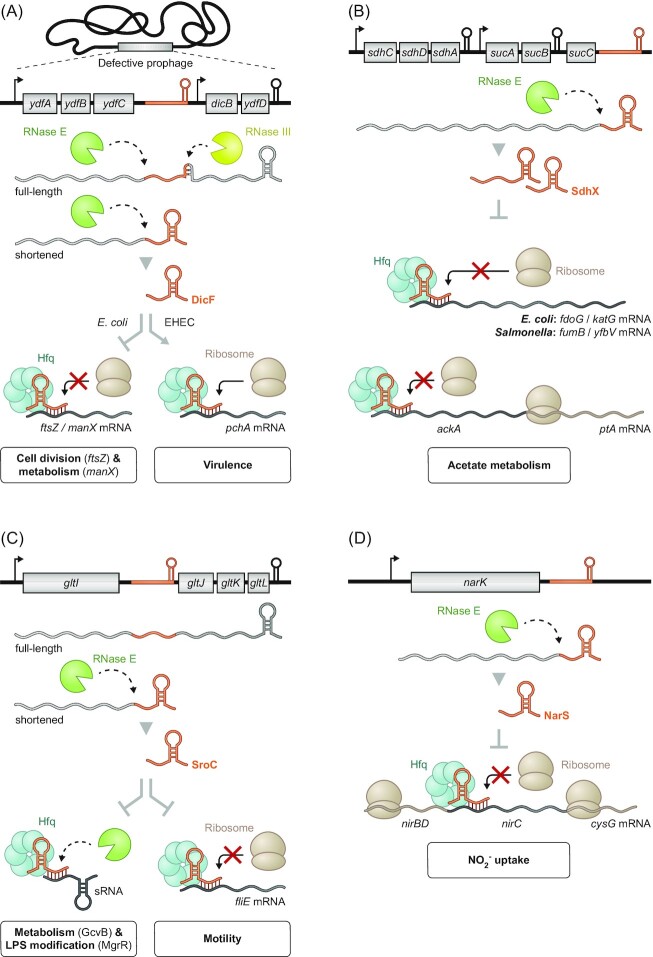
Enterobacterial 3′ UTR-derived sRNAs are involved in diverse pathways. **(A)** The DicF sRNA stems from inside the *E. coli ydfABC-dicF-dicB-ydfD* operon mRNA that is transcribed from a defective prophage region. Processing of the mRNA by both RNase E and RNase III yields DicF, which in turn is involved in the regulation of cell division and metabolism. Additionally, in EHEC, DicF is also important for the regulation of the pathogen's virulence by upregulating the transcriptional activator PchA. **(B)** RNase E-dependent processing at the 3′ end of the *sdhCDAB-sucABCD* mRNA generates the sRNA SdhX, which selectively acts on *ackA* of the bicistronic *ackA-ptA* operon to regulate acetate metabolism. Additionally, SdhX exhibits divergent targetomes in *E. coli* and *Salmonella*. **(C)**Premature transcriptional termination of the *gltIJKL* operon and subsequent RNase E-mediated cleavage frees the sRNA SroC from its parental operon. While SroC is involved in regulating motility by repressing translation of *fliE*, SroC has an expanded regulatory capacity through sponging the sRNAs GcvB and MgrR to further affect metabolism and LPS modification, respectively. **(D)** NarS is processed off the 3′ end of *narK* mRNA by RNase E. By selectively inhibiting translation of *nirC* as part of the *nirBDC-cysG* operon, this sRNA works synergistically with the NarK protein to fine-tune nitrite uptake.

Though not initially realized as such, DicF was the first 3′ UTR-derived sRNA to be characterized (Fig. 4A). It originates from the 3′ UTR of *ydfC* in the *ydfABC-dicF-dicB-ydfD* operon (historically named the ‘*dicBF* operon’), which is located in a defective lambdoid prophage in the *E. coli* K-12 genome (Bouché and Bouché 1989). The functional 53-nt DicF sRNA is generated via early Rho-independent transcription termination followed by RNase E-dependent processing at the 5′ end (Faubladier *et al*. 1990). An alternative 3′ end of DicF is generated by RNase III-mediated processing of the full-length *dicBF* RNA, releasing a 72-nt DicF species, which represents an intra-operonic sRNA rather than a true 3′ UTR-derived one (Faubladier *et al*. 1990, Balasubramanian *et al*. 2016).

DicF was originally described as a genomic element that blocked cell division in *E. coli*, inhibiting the synthesis of the essential cell division protein FtsZ (Tétart *et al*. 1992). More recently, it was shown that DicF is an Hfq-dependent sRNA that inhibits not only the translation of the *ftsZ* mRNA but also of additional mRNAs (*manX*,*pykA* and *xylR*) with functions in metabolism (Balasubramanian *et al*. 2016). This also involves a noncanonical mechanism whereby DicF may repress *manX* translation indirectly, through loading Hfq onto the RBS of this mRNA (Azam and Vanderpool 2018).

Furthermore, DicF was shown to function as a specific translational activator in enterohemorrhagic *E. coli*. Here, the sRNA competes with the formation of an intrinsic inhibitory structure in the *pchA* mRNA under microaerobic conditions. By upregulating the transcriptional regulator PchA, DicF indirectly activates synthesis of the virulence-associated type 3 secretion system of this pathogen (Melson and Kendall 2019, Murashko and Lin-Chao 2017). Together, these examples illustrate that the physiological impact of the 3′ UTR-derived DicF sRNA extends well beyond the initially described inhibition of cell division.

## SdhX: coordination with the TCA cycle

CpxQ, described further earlier, is the released 3′ UTR of a monocistronic mRNA. By contrast, the sRNA SdhX (originally known as RybD; Zhang *et al*. 2003) is generated from the very end of the large *sdhCDAB-sucABCD* operon mRNA (Zhang *et al*. 2003, Cronan and Laporte 2005) (Fig. 4B). Encoding key proteins of the tricarboxylic acid cycle (TCA cycle), the *sdh-suc* operon is subject to complex regulation by TFs and sRNAs (Park *et al*. 1997, Nam *et al*. 2005). Successive RNase E cleavages generate two variants of SdhX: one ∼101 nt and the other only ∼38 nt in length (Miyakoshi *et al*. 2019). Both of these variants contain the conserved seed region of SdhX through which this sRNA recognizes its major target, the mRNA of acetate kinase AckA (De Mets *et al*.^2019^, Miyakoshi *et al*. 2019). Intriguingly, the SdhX-mediated translational repression affects only *ackA* but not the other gene of the bicistronic *ackA-pta* operon. The *pta* gene is also involved in acetate metabolism, and the discordant regulation by SdhX may have evolved to selectively increase the accumulation of the signaling molecule acetyl phosphate (De Mets *et al*. 2019, Miyakoshi *et al*. 2019).

The targetome of SdhX is likely to be much larger, yet also species-specific. Additional mRNA targets in *Salmonella* are *fumB* and *yfbV* (Miyakoshi *et al*. 2019), whereas in *E. coli* SdhX also represses the *fdoG* and *katG* mRNAs (De Mets *et al*. 2019, Melamed *et al*. 2016, 2020). These differences are the result of single-nucleotide changes between the respective *E. coli* and *Salmonella* genes; whether they are just random mutations or reflect different physiological needs of these two species is unknown. The conserved regulation of *ackA* by SdhX, however, is a paramount example of how the transcript of a major metabolic operon (i.e. the TCA cycle) post-transcriptionally influences another metabolic operon (i.e. acetate metabolism) via the activity of a 3′ UTR-derived sRNA. In addition, the high variability of the SdhX sequence outside the short seed (Miyakoshi *et al*. 2019) further argues that mRNA 3′ UTRs are a playground for the evolution of regulatory sRNAs.

## SroC: sponging another sRNA

While the hitherto described examples regulate mRNAs, the primary target of SroC is different: SroC acts as a regulatory sponge of another sRNA (Fig. 4C). Similar to the biogenesis of DicF, SroC is produced from within the *gltIJKL* operon such that early Rho-independent termination yields a monocistronic *gltI* (a.k.a. *ybeJ*) transcript, processing of which leaves the final 163-nt SroC species (Vogel *et al*. 2003, Miyakoshi *et al*. 2015a). SroC acts by Hfq-dependent base pairing to accelerate the decay of GcvB, the latter of which is a well-characterized sRNA that represses ∼1% of all *E. coli* and *Salmonella* genes (Urbanowski *et al*. 2000, Pulvermacher *et al*. 2009, Sharma *et al*. 2007, 2011, Busi *et al*. 2010, Vanderpool 2011, Stauffer and Stauffer 2012, Wright *et al*. 2013, Yang *et al*. 2014, Miyakoshi *et al*. 2022). Many of the mRNAs repressed by GcvB function in amino acid transport and metabolism. Intriguingly, since the *gltIJKL* mRNA itself is a target of GcvB, the SroC sponge seems to enable both a positive feedback loop to activate its parental mRNA in *cis*, while also activating many *trans*-encoded mRNAs in the same pathway. Physiologically, loss of SroC impairs bacterial growth when peptides are the sole carbon and nitrogen sources (Miyakoshi *et al*. 2015a).

Interestingly, SroC sponges more than one Hfq-dependent sRNA. In *E. coli*, SroC was also shown to downregulate the MgrR sRNA, thereby alleviating MgrB-mediated translational repression of the LPS modification enzyme EptB (Acuña *et al*. 2016). Furthermore, classic sRNA activity on mRNAs has also been reported, showing that SroC negatively regulates the *flhBAE* and *fliE* mRNAs through direct base pairing and thus flagella synthesis in *Salmonella* (Fuentes *et al*. 2015). Therefore, SroC—once considered ‘a putative processed fragment of the *ybeJ–gltJ* spacer’ (Vogel *et al*. 2003)—has emerged as a multi-facetted regulator of processes as diverse as metabolism, motility and surface modification.

## NarS: avoiding self-poisoning

As mentioned earlier, our recognition of processed 3′ UTRs as potential sRNAs grew with their detection as stable fragments in northern blot or RNA-seq analyses. Such detection requires the parental mRNA to be expressed under the condition of the assay, but many bacterial genes are only transcribed under specific growth conditions. A case in point is the NarS sRNA (Wang *et al*. 2020), whose parental gene is only activated by FNR or NarL under anaerobic growth or high levels of nitrite, respectively, to produce the nitrate transport protein NarK (Kolesnikow *et al*. 1992, Kröger *et al*. 2013).

The mature 63-nt NarS sRNA (Fig. 4D) spans a little more than the 3′ UTR of *narK*, and is conserved in a subclade of the Enterobacteriaceae. NarS appears to have one main activity, which is to repress the *nirC* mRNA encoding a major nitrite importer. A simple working model postulates that the NarK protein will both import extracellular nitrate for nitrate reduction and export the product nitrite. Concomitantly, the *narK*-derived sRNA NarS functions to prevent expression of the nitrite importer NirC to limit uptake of excessive nitrite from the environment in order to avoid self-poisoning. Questions remain with respect to the molecular mechanism of target regulation, for example, why and how the NarS sRNA very selectively regulates only the *nirC* cistron of the much longer *nirBDC-cysG* operon mRNA (Wang *et al*. 2020).

## MalH: helping to use the right carbon source through porin regulation

Continuing on expression under very specific conditions, the 104 nt long MalH sRNA from the 3′ end of the maltose uptake operon *malEFG* mRNA (Iosub *et al*. 2021) shows expression that is as selective as that of NarS (Wang *et al*. 2020). This *mal* operon is strongly suppressed in the presence of glucose, but when glucose becomes scarce or maltose is present, its expression is activated by the TF MalT to ensure maltose uptake. Under these latter conditions, MalH accumulates and negatively regulates the mRNAs of the abundant OmpA and OmpC porins. Additionally, high levels of MalH lead to a reduction of the σ^E^-dependent sRNA MicA, which is expected to lift the known MicA-mediated repression of the mRNA of the maltoporin LamB (Bossi and Figueroa-Bossi 2007, Gogol *et al*. 2011, Wang *et al*. 1997). While the exact mechanisms of MalH, especially how it derepresses LamB synthesis, are yet to be resolved, the current working model suggests that this sRNA helps to shift carbon metabolism to maltose when *E. coli* bacteria run out of their favored carbon source, glucose. Interestingly, MalH shares the same genomic location as MicX found in *V. cholerae*. However, thus far no common targets are known for both sRNAs and MicX is transcribed from its own promoter, in contrast to the biogenesis of MalH (Davis and Waldor 2007). This makes the MalH–MicX pair an interesting example of the divergent evolution of an sRNA encoded in two bacteria living different lifestyles.

## FarS: switching fatty acid metabolism

**Figure 5.**
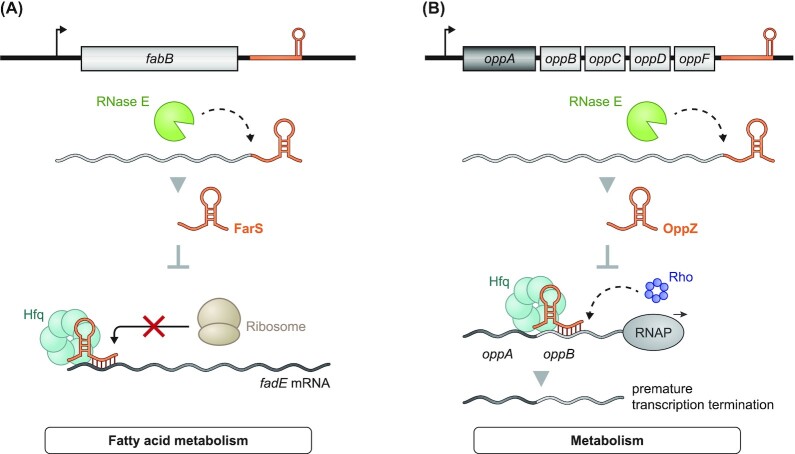
3′ UTR-derived sRNAs in *V. cholerae*. **(A)** RNase E-mediated processing yields the sRNA FarS from its parental mRNA *fabB*. Through inhibition of *fadE* translation, FarS is involved in the regulation of the fatty acid metabolism of the pathogen. **(B)** The 3′ end of the *oppABCDF* operon contains the sRNA OppZ, which is generated through RNase E processing. OppZ acts self-regulating by initiating Rho-dependent transcription termination downstream of *oppA*. This leads to the fine-tuning of the protein stoichiometry of the OppABCDF system.

*V. cholerae* is a human pathogen in which 3′ UTR-derived sRNAs seem to be very prominent. A global Hfq coimmunoprecipitation study (Huber *et al*. 2020) found about half of all sRNAs that were enriched with this RNA chaperone to be processed mRNA 3′ fragments, of which FarS (Fig. 5A) was studied in detail. RNase E-mediated mRNA turnover generates FarS from *fabB*, which encodes the first enzyme in the fatty acid synthesis pathway. While FabB promotes the production of acyl-ACP from acyl-CoA, the associated FarS sRNA together with Hfq translationally represses the synthesis of FadE, which is the first enzyme in the opposing β-oxidation cycle that produces acetyl-CoA from long chain acyl-CoA. On top of that, the *fabB* and *fadE* genes are antagonistically regulated by the same major TF of fatty acid metabolism, FadR. Hence, the FarS sRNA and the FadR regulatory protein constitute a mixed feed-forward loop regulating the transition between fatty acid biosynthesis and degradation (Huber *et al*. 2020).

## OppZ: enabling gene autoregulation

Staying with 3′ UTR-derived sRNAs of *V. cholerae*, functional characterization of OppZ has helped to identify a new sRNA-dependent mechanism: autoregulation of the parental gene (Hoyos *et al*. 2020). The 52-nt OppZ sRNA is generated from the 3′ end of the *oppABCDF* operon in an RNase E-dependent fashion, and its stability and function rely on Hfq (Fig. 5B). Surprisingly, OppZ was found to bind its own operon mRNA in the IGR between *oppA* and *oppB*, leading to downregulation of translation of all encoded proteins except OppA. This further caused downregulation of OppZ itself. However, stability of the *oppB* mRNA was not affected by OppZ, indicating that this sRNA acted by terminating transcription rather than inducing transcript decay. Mutational studies confirmed this hypothesis, revealing that translational inhibition of *oppB* by OppZ causes Rho-dependent transcription termination and with this downregulation of *oppBCDF-oppZ* without affecting *oppA* (Hoyos *et al*. 2020).

Negative autoregulation is common among TFs, which often control their own expression by blocking their own promoter (Rosenfeld *et al*. 2002). In contrast, OppZ and the *V. cholerae* CarZ sRNA (identified in the same study) represent the first examples of sRNAs that use this principle to control their own expression (Hoyos *et al*. 2020). Autoregulation via 3′ UTR-derived sRNAs seems to be particularly suited for polycistronic mRNAs such as *oppABCDF* since it requires the operon to be fully transcribed for the negative feedback mechanism to kick in, ensuring balanced protein synthesis and enabling discordant expression of the operon's individual genes.

## RaiZ: moving to ProQ

**Figure 6.**
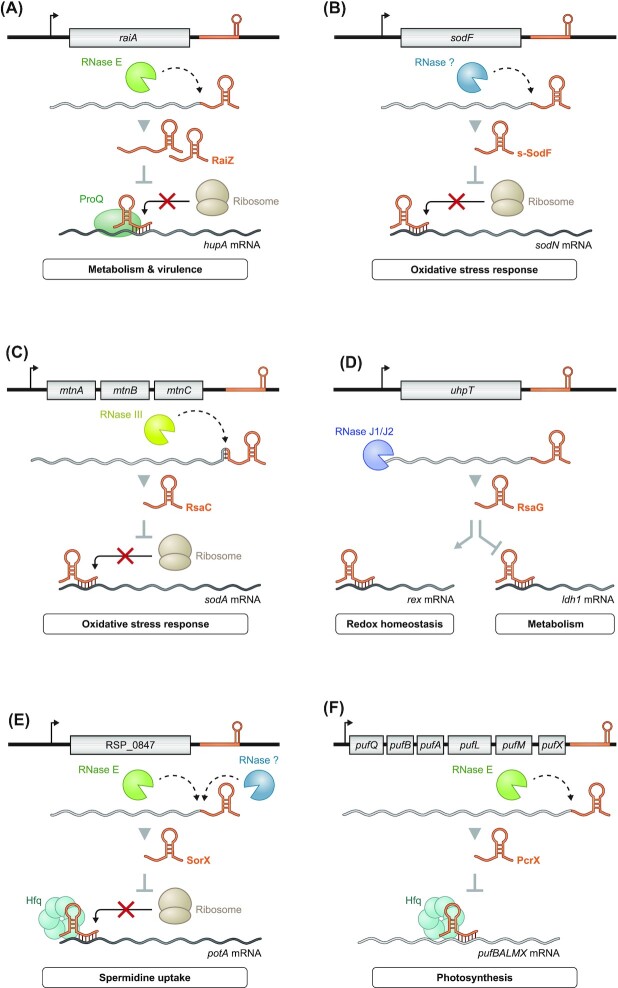
Further examples of 3′ UTR-derived sRNA in diverse organisms. **(A)** The sRNA RaiZ derived from the 3′ end of the *raiA* mRNA acts in ProQ-dependent manner by suppressing the globally acting DNA-binding protein *hupA* affecting metabolism and virulence of *Salmonella* and *E. coli*. **(B)** Processing of the *sodF* mRNA by an unknown ribonuclease gives rise to the s-SodF sRNA. The sRNA allows *S. coelicolor* to switch the superoxide dismutase (SOD) in response to oxidative stress under Ni-limited conditions. **(C)** In *Staphylococcus*, the sRNA RsaC is processed via RNase III from the *mntABC* operon at low Mn^2+^ levels. Under these conditions, RsaC represses the Mn-dependent SOD *sodA* allowing an efficient response against oxidative stress. **(D)** The sRNA RsaG is generated via 5′ → 3′ degradation by RNase J1/J2 of the full-length *uhpT* mRNA. Binding of RsaG to its target can lead to either stabilization or degradation of the bound mRNAs. In case of the former, RsaG binds the *rex* mRNA, which is a redox regulator. In case of the latter, binding of RsaG accelerates degradation of the lactate dehydrogenase (*ldh1*) mRNA and thus impacts metabolism. **(E)** The sRNA SorX is part of the 3′ end of RSP_0847 in *R. sphaeroides* and generated through an unknown RNase combined with RNase E. The released sRNA inhibits spermidine uptake through translational inhibition of the polyamine uptake transporter *potA* and thus supports fighting off oxidative stress. **(F)** The sRNA PcrX represents the 3′ end of the photosynthesis complex *pufQBALMX* operon and is generated via RNase E. PcrX acts self-regulating by destabilizing its parental mRNA through a yet unknown mechanism.

The protein ProQ has recently been established as the third globally acting sRNA-related RBP of Gram-negative bacteria, after CsrA and Hfq (Smirnov *et al*. 2016). While initially described in *Salmonella* as a target of Hfq (Chao *et al*. 2012), the RaiZ sRNA (Fig. 6A) has since been recovered in association with ProQ in several independent studies (Smirnov *et al*. 2016, Holmqvist *et al*. 2018, Melamed *et al*. 2020). Moreover, RaiZ requires ProQ rather than Hfq for its intracellular stability, and is therefore now considered a ProQ-dependent sRNA (Smirnov *et al*. 2016, Smirnov *et al*. 2017b). Its biogenesis, however, follows the same pattern as Hfq-dependent 3′ UTR-derived sRNAs: RNase E degrades the mRNA of the translational inhibitor and ribosome stability factor RaiA, resulting in the accumulation of two forms (∼160 and ∼122 nt) of the RaiZ sRNA (Kröger *et al*. 2013, Smirnov *et al*. 2017b).

The first RaiZ target to be identified was the *hupA* mRNA encoding the α-subunit of histone-like protein HU. Mechanistically, RaiZ inhibits *hupA* translation, forming an imperfect RNA duplex long enough to attract RNase III for mRNA cleavage (Smirnov *et al*. 2017b). As such, knowledge of RaiZ enabled the first mechanistic investigation of mRNA repression *in trans* by a ProQ-dependent sRNA.

It is yet to be understood why the 3′ UTR of a translation-associated protein would repress the synthesis of a major DNA-binding protein with roles in controlling central metabolism and virulence. Interestingly, RaiZ is also upregulated during the early stages of *Salmonella* infection of HeLa cells (Westermann *et al*. 2019). Thus, RaiZ may help the bacteria to adjust global transcription to changing environments, while the protein encoded by the *raiA* mRNA acts similarly to globally adjust translation rates. These activities may be conserved as an independent screen in *E. coli* confirmed *hupA* mRNA as a top interactor of RaiZ (Melamed *et al*. 2020). This screen further identified additional putative targets of RaiZ such as the *lpp* mRNA and the abundant ProQ-associated sRNA RyfD. It may be through the investigation of these additional targets that we will fully understand the physiological implications of RaiZ activity.

## s-SodF: switching to the better enzyme

The 90-nt s-SodF sRNA is a particularly clear example of how bacteria use released 3′ UTR fragments as regulatory mRNA switches (Kim *et al*. 2014) (Fig. 6B). Enzymes of the superoxide dismutase (SOD) family generally protect bacteria from oxygen-derived superoxides and resulting oxidative damage. Such is the case in *S. coelicolor*, which possesses two mutually exclusively expressed SOD proteins, a Ni-dependent SOD (*sodN*) and an Fe-dependent SOD (*sodF*) (Chung *et al*. 1999). Their mutually exclusive expression is mainly regulated by the TF Nur, which represses the *sodF* gene and activates the *sodN* gene in the presence of nickel (Chung *et al*.^1999^, Ahn *et al*. 2006).

This transcriptional switch is elegantly complemented by the s-SodF sRNA, which works at the post-transcriptional level in the opposite direction. When the *sodF* mRNA is expressed, a yet unknown nuclease releases its 3′ UTR in the form of s-SodF. The s-SodF sRNA forms a 19-bp RNA duplex with the 5′ region of the *sodN* mRNA, triggering rapid mRNA decay. The result is a mixed feed-forward loop composed of a TF and two cross-talking mRNAs that should facilitate rapid activation of the expression of the Fe-dependent SodF as nickel becomes scarce (Kim *et al*. 2014). Whether s-SodF requires other factors for function is unknown, but the clarity of the system begs for a genetic screen, which may eventually find a currently elusive sRNA-related RBP in the genus *Streptomyces*.

## RsaC: keeping things running during manganese limitation

The human pathogen *S. aureus* has been a model species for sRNA screens in Gram-positive bacteria. An early screen described the RsaC sRNA (Geissmann *et al*. 2009), and a more recent one found RsaC to be upregulated *in vivo* in a mouse model of osteomyelitis (Szafranska *et al*. 2014). RsaC, which is conserved in *S. aureus* and *Staphylococcus argenteus*, is unusually long. It ranges in length from 584 nt to 1116 nt due to repeats in its 5′ region (Lalaouna *et al*. 2019) and is generated by RNase III-dependent cleavage from the *mntABC* operon mRNA (Lioliou *et al*. 2012) (Fig. 6C). This operon encodes a major importer of manganese. When manganese is plentiful, the TF MntR represses the *mntABC* genes, resulting in low levels of RsaC (Lieser *et al*. 2003).

All of this makes sense in light of the function of SodA, whose mRNA has been identified as the main target of RsaC. SodA is a Mn-dependent SOD, explaining why RsaC represses it under Mn-limiting conditions. Consistently, while RsaC represses SodA synthesis under Mn-limiting conditions, the alternative SOD SodM, which is active when loaded with either Mn^2+^ or Fe^2+^, shows increased expression under these conditions (Lalaouna *et al*. 2019). Of note, sequestration of metal ions such as Mn^2+^ represents an important host defense mechanism to limit pathogen growth. Thus, RsaC acts at the interface of the *S. aureus* oxidative stress response and host nutritional immunity.

## RsaG: a jack of all trades

The global screen that identified RsaC in *S. aureus* also discovered RsaG, an sRNA derived from the mRNA 3′ end of hexose phosphate antiporter UhpT (Geissmann *et al*. 2009). The *uhpT*-*rsaG* operon is induced when coculturing this extracellular pathogen with mucin-producing eukaryotic cells, when internalized or—most simply—when glucose-6-phosphate (G6P) is present in the growth medium (Bronesky *et al*. 2019, Desgranges *et al*. 2021). Invariably, transcriptional activation of RsaG's parental mRNA depends on the G6P-sensing two-component system HptRS (Desgranges *et al*. 2021, Park *et al*. 2015). Remarkably, RsaG is generated via 5′ → 3′ degradation by the exoribonucleases J1/J2 and as such represents the first example for this route of sRNA biogenesis. This process requires the full-length *uhpT*-*rseG* RNA and possibly a hairpin structure at the 5′ end of RsaG, which is thought to block the exonucleolytic activities of the RNases to yield the mature sRNA (Desgranges *et al*. 2021).

With respect to function, RsaG is a great example of the diversity of molecular mechanisms utilized by sRNAs: it sequesters the RBS of some of its targets, accelerating their degradation, while also stabilizing other targets (Desgranges *et al*. 2021). Regarding the latter, binding of RsaG to the *rex* mRNA increases the transcripts half-life of this global redox regulator. Of note, Rex is a repressor of the lactate-dehydrogenase *ldh1* gene, whose mRNA is downregulated by RsaG (Fig. 6D) (Pagels *et al*. 2010, Desgranges *et al*. 2021). Moreover, RsaG can interact with the sRNA RsaI, an additional negative regulator of *ldh1* (Bronesky *et al*. 2019, Desgranges *et al*. 2021). The consequences of this sRNA–sRNA interaction are not fully understood. Still, this complex targetome suggests that RsaG is a versatile regulator, contributing to the modulation of redox homeostasis and metabolism upon changing environmental conditions in *S. aureus*. Other putative targets of RsaG include mRNAs involved in virulence and biofilm formation (Desgranges *et al*. 2021).

## SorX: combatting oxidative stress

Similar to the earlier examples from *S. coelicolor* and *S. aureus*, the photoheterotrophic bacterium *R. sphaeroides* also employs a 3′ UTR-derived sRNA, called SorX (Fig. 6E), to combat oxidative stress. This Hfq-dependent sRNA responds to, and confers resistance to, stress by singlet oxygen and organic hydroperoxides (Peng *et al*. 2016). It is cleaved off the 3′ end of *RSP_0847* (encoding an OmpR-type TF) by an unknown nuclease to generate a 116-nt pre-SorX RNA, which is subsequently processed by RNase E to release the mature 75-nt SorX sRNA. In contrast to *E. coli*, where polyamines such as spermidine are thought to be beneficial under oxidative stress conditions (Rhee *et al*. 2007), spermidine exacerbates the detrimental effects of reactive oxygen species in *R. sphaeroides* (Peng *et al*. 2016). SorX counteracts this toxicity by reducing spermidine uptake via translation inhibition of *potA*, which is part of the PotABCD polyamine transporter.

## PcrX: balancing photosynthesis

*R. sphaeroides* is able to perform both aerobic respiration and anoxygenic photosynthesis, which need to be tightly controlled since bacteriochlorophyll can generate harmful singlet oxygen in presence of light and oxygen (Glaeser *et al*. 2011). Photosynthesis requires proteins encoded by the *pufQBALMX* operon. Intriguingly, this polycistronic mRNA also harbors a 3′ UTR-derived sRNA, termed PcrX (Fig. 6F), which is generated by RNase E-dependent cleavage (Eisenhardt *et al*. 2018). PcrX destabilizes the 3′ region (*pufLMX*) of its parental transcript in an Hfq-dependent manner, probably by targeting a region within *pufX*, which subsequently leads to a decrease in functional photosynthetic complex levels. This putative mechanism exemplifies how bacteria might use 3′ UTR-derived sRNAs to maintain steady levels of important complexes.

## Common regulatory networks of 3′ UTR-derived sRNAs

**Figure 7.**
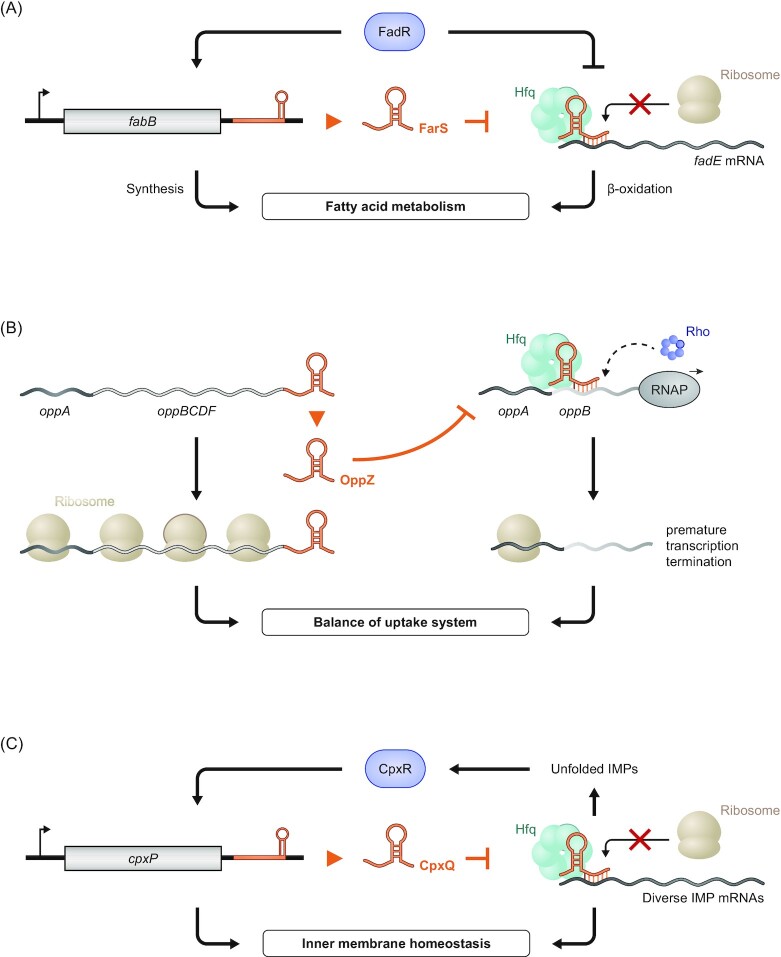
Common regulatory networks of 3′ UTR-derived sRNAs. **(A)** In *V. cholerae*, the transcription factor FadR activates the expression of FarS and its parental mRNA *fabB*, of which the latter is involved in fatty acid synthesis. Simultaneously, FadR and FarS repress *fadE* expression that is part of the opposing β-oxidation pathway for fatty acids. Thus, this mixed feed-forward loop enables an efficient fatty acid metabolism. **(B)**The autoregulatory sRNA OppZ ensures a proper balance of the different proteins of the Opp peptide uptake system in *V. cholerae*. Through causing premature transcription termination of its parental mRNA, for all but the peptide-binding protein OppA, OppZ limits expression of *oppBCDF* as well as itself. **(C)** Mixed feed-forward loops can also be involved in the regulation of stress. As exemplified by the CpxR-dependent protein CpxP and sRNA CpxQ of the Enterobacteriales. Through translational inhibtion of diverse IMPs, CpxQ reduces the CpxR-mediated transcription of CpxP as well as the sRNA itself. Both components of the Cpx pathway ensure a reduction of misfolded IMPs and thus alleviate IM stress.

Most of the well-studied examples of 3′ UTR-derived sRNAs described earlier are involved in the regulation of various metabolic pathways. They often seem to do so by acting on a single main target, in contrast to the common multi-target regulation by classic sRNAs (Hör *et al*. 2020a). Additionally, the preferred regulatory circuit of 3′ UTR-derived sRNAs involved in metabolism seems to be a mixed feed-forward loop, with a TF that regulates the levels of both the sRNA and its target. This type of regulation is especially well suited to buffer residual transcriptional noise through additional regulation at the post-transcriptional level, thereby leading to tighter control of expression (Nitzan *et al*. 2017). In the case of metabolic regulons, this tight control is of particular importance as many mRNAs act in opposing pathways to those regulated by the sRNAs they are carrying. FarS is an exceptionally clear example of this relationship: The TF FadR upregulates the fatty acid synthesis gene *fabB*, and concomitantly downregulates *fadE*, which is involved in the opposing β-oxidation pathway (Fig. 7A). The *fabB*-derived FarS sRNA completes the feed-forward loop through a post-transcriptional repression of *fadE*, thereby ensuring that fatty acids are not being synthesized and degraded at the same time (Quax *et al*. 2013, Huber *et al*. 2020).

Another important feature in the regulation of metabolic pathways is to retain optimal stoichiometry between different subunits of complexes or between enzymes of the same pathway. The structure and translational efficiency of polycistronic mRNAs in bacteria are known to be key to maintaining stoichiometry (Li *et al*. 2014, Burkhardt *et al*. 2017). Similarly, 3′ UTR-derived sRNAs can regulate stoichiometry by forming a negative feedback loop leading to repression of only a part of their parental polycistronic mRNAs, as shown for OppZ in *V. cholerae* (Fig. 7B). Here, the sRNA does not regulate *oppA*, the first cistron of the transcript, which encodes for a stand-alone periplasmic protein needed in high quantity. Instead, OppZ downregulates the downstream *oppBCDF* (and thereby itself), whose protein products form a transporter complex for oligopeptides. Given that the *oppABCDF* transcript is exclusively regulated upstream of *oppA*, regulation in *cis* via a 3′ UTR-derived sRNA ensures protein copy numbers independent of other regulators. The authors of this study argue that 3′ UTR-derived autoregulation is best for this purpose, as the OppZ sRNA being generated by ribonucleolytic cleavage comes at a 1:1 stoichiometry with its sole target, the *oppBCDF* mRNA (Hoyos *et al*. 2020).

In a mixed circuit, two regulators deriving from the same transcript are involved in the same or complementary pathways (Nitzan *et al*. 2017). This type of regulation is particularly well suited for stress response genes, as transcription of a single RNA leads to a fast, multi-pronged answer to the stress. There are two types of mixed circuits involving sRNAs: dual-function sRNAs, such as SgrS, encode a regulatory protein that functions in the same pathway as its parental sRNA (Wadler and Vanderpool 2007, Raina *et al*. 2018). The other type involves 3′ UTR-derived sRNAs, of which the best-studied example is CpxQ. This sRNA together with the CpxP protein from its parental mRNA forms a mixed circuit to alleviate IM stress (Chao and Vogel 2016, Grabowicz *et al*. 2016). While CpxP and CpxQ work coordinately, CpxP acts on IMPs at the post-translational level, whereas CpxQ downregulates IMP synthesis at the translational level (Fig. 7C).

Overall, 3′ UTR-derived sRNAs mainly seem to be specialized regulators involved in the maintenance of currently needed or favored metabolic processes, which typically depends on the availability of nutrients or trace elements. Yet, global methods such as RIL-seq (discussed later) are challenging this view by identifying a plethora of additional targets of known 3′ UTR-derived sRNAs (Melamed *et al*. 2016), questioning our current understanding of the regulatory roles of these sRNAs.

## Using global methods to predict 3′ UTR-derived sRNAs and their targets

Going forward, it will be useful to employ global methods to comprehensively search for 3′ UTR-derived sRNAs. This will be of special importance for understudied species that are evolutionarily distant from model γ-proteobacteria such as *E. coli*,*S. enterica* and *V. cholerae* (Hör *et al*. 2018).

**Figure 8.**
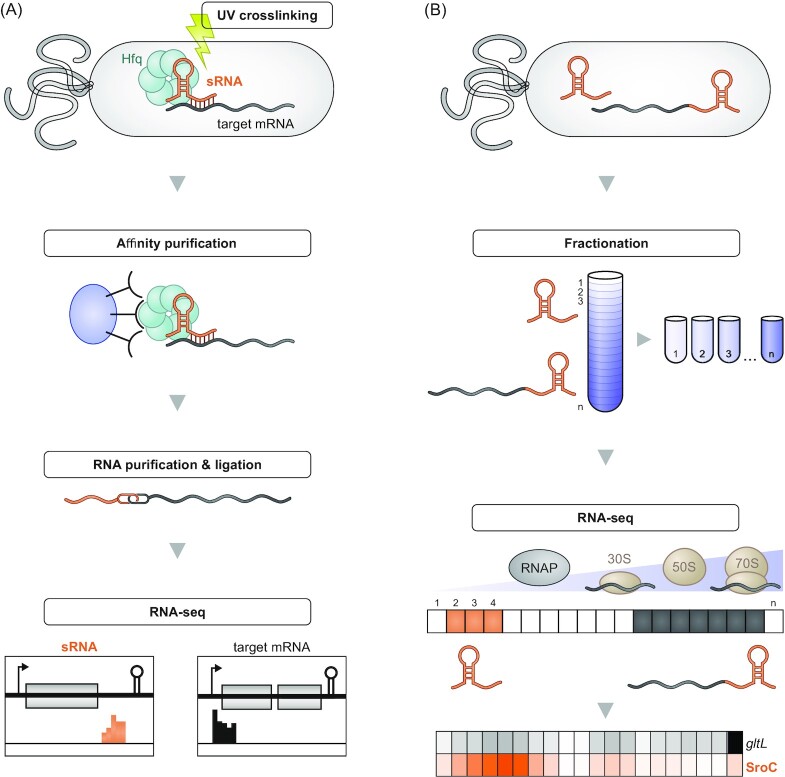
Global methods to uncover 3′ UTR-derived sRNAs and their targets. **(A)** A schematic overview for the workflow of methods relying on proximity ligation to identify sRNAs and their targets such as RIL-seq or CLASH. Split read mapping of the ligated RNA products allows the identification of the interacting transcripts and can thus globally uncover interactions between 3′ UTRs of genes and potential targets. **(B)** Grad-seq can enable the identification of novel 3′ UTR-derived sRNAs in a global manner. Fractionation of a cell lysate and subsequent sequencing can uncover differential migration patterns of a parental mRNA and its 3′ UTR and thus indicate an additional function of the latter as an sRNA, as shown for the example of SroC (data acquired from Smirnov *et al*. 2016).

RIP-seq and the related CLIP-seq technique can be exploited to globally identify a pool of potential 3′ UTR-derived sRNAs associated with an RBP of interest. Yet, these methods cannot easily distinguish between the 3′ UTR of a CDS and a functionally independent sRNA derived from the same sequence. To circumvent this issue, RIL-seq and CLASH add a proximity ligation step to the CLIP-seq protocol, which enables the identification of functional RNA–RNA interactions *in vivo* (Hör and Vogel 2017, Waters *et al*. 2017, Iosub *et al*. 2020, Matera *et al*. 2022, Melamed *et al*. 2016, 2018, 2020) (Fig.   8A). RIL-seq in *E. coli*, for instance, revealed two hitherto unknown 3′ UTR-derived sRNAs, PspH and GadF, to be a sponge of the sRNA Spot 42 and a regulator of acid stress response genes, respectively—interactions that are easily overlooked by conventional target searches (Melamed *et al*. 2016). Most recently, the development of a new machine learning-based algorithm helped to discover several additional 3′ UTR-derived sRNAs and their targets in *E. coli* RIL-seq data (Bar *et al*. 2021).

Where a major sRNA-related RBP is unknown, as is the case with most Gram-positive species, GRIL-seq and its variant Hi-GRIL-seq can be used in a similar way as RIL-seq or CLASH (Han *et al*. 2016, Zhang *et al*. 2017). In GRIL-seq, an RNA ligase is expressed *in vivo*, causing proximity ligation between interacting RNAs. Following sequencing, the chimeric reads containing ligated transcripts can be searched for 3′ UTRs that interact with other mRNAs or sRNAs. In *Pseudomonas aeruginosa*, this led to the identification of SkatA, a 3′ UTR-derived sRNA that functions as a sponge of the PrrF1 sRNA involved in iron regulation (Han *et al*. 2016, Zhang *et al*. 2017). Intriguingly, expression of SkatA protected its parental mRNA *katA* from PrrF1-dependent downregulation, suggesting a mechanism that ensures basal expression of the catalase protein encoded by *katA*.

A recent modification of the GRIL-seq approach has focused on identifying novel sRNAs that interact with an mRNA of interest. Using this so-called ‘reverse GRIL-seq’ approach, analysis of the interaction partners of the *rpoS* mRNA in *V. cholerae* identified Vcr043 as a novel activator of this important mRNA (Han and Lory 2021).

Alternatively, a combined approach identifying processed RNA 5′ ends via dRNA-seq or Cappable-Seq (Sharma *et al*. 2010, Ettwiller *et al*. 2016) and RNA 3′ ends via term-seq (Dar *et al*. 2016) or similar methods (Shishkin *et al*. 2015, Yan *et al*. 2018, Ju *et al*. 2019, Fuchs *et al*. 2021) is able to directly predict 3′ UTR-derived sRNAs with single-nucleotide precision. Such a two-pronged approach was recently applied to map both 5′ UTRs and 3′ UTRs in the human gastrointestinal pathogen *Clostridioides difficile* and led to the identification of 18 hitherto unknown 3′ UTR-derived sRNAs (Fuchs *et al*. 2021), illustrating the strength of combining synergistic methods.

Finally, full-length single molecule sequencing methods such as PacBio and Oxford Nanopore sequencing—the latter of which even allows direct RNA sequencing—are expected to bolster functional transcript discovery without the need for special sample preparation. For all of the mentioned methods, downstream verification of the existence and targets of putative 3′ UTR-derived sRNAs should be performed via standard techniques such as northern blotting or extrachromosomal expression.

## Using grad-seq to predict functional 3′ UTR fragments

Global transcriptomics via RNA-seq allows us to detect transcripts from any region of an organism's genome, i.e., noncoding as well as coding transcripts including transcribed 5′ and 3′ UTRs. However, the currently dominant method of short read sequencing used in standard RNA-seq cannot easily tell a functionally independent 3′ UTR-derived sRNA apart from the regular 3′ end of its much longer mRNA. This caveat necessitates the development of alternative transcriptomic methods that enable the identification of functional classes of transcripts rather than looking at the mere presence of transcripts.

In this regard, Grad-seq (Smirnov *et al*. 2016, 2017a, Hör *et al*. 2020b,c, Gerovac *et al*. 2021a,b, Hör and Vogel^2021^, Lamm-Schmidt *et al*. 2021, Riediger *et al*. 2021) is a promising technique for the identification of new 3′ UTR-derived sRNAs in both model and understudied or genetically intractable organisms. In Grad-seq, the soluble molecules of a bacterial lysate are separated on a glycerol gradient according to their weight and shape. Subsequent fractionation of the gradient followed by RNA-seq of each fraction then reflects the sedimentation of each transcript (Gerovac *et al*. 2021a). If a 3′ UTR-derived sRNA has an independent function, it can be expected to be involved in different interactions than its parental mRNA (i.e. it binds its target RNA and/or interacting RBP instead of the ribosome), meaning it will sediment in different parts of the gradient, thereby facilitating its identification (Fig. 8B). This functional relationship was recently exploited to identify a 3′ UTR-derived sRNA in the giant bacteriophage ΦKZ, highlighting the power of Grad-seq for sRNA discovery (Gerovac *et al*. 2021b).

## Conclusion and outlook

**Figure 9.**
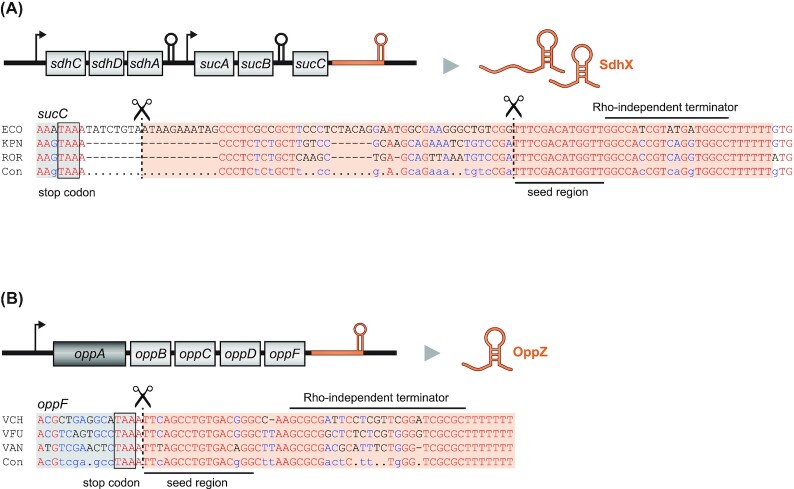
3′ UTR-derived sRNAs are a conserved feature of their parental mRNA. **(A)** Sequence alignment for *sucC* and the sRNA SdhX (ECO: *E. coli*; KPN: *Klebsiella pneumoniae*; ROR: *Raoultella ornithinolytica*; Con: consensus sequence).**(B)** Sequence alignment for *oppF* and the sRNA OppZ (VCH: *V. cholerae*; VFU: *V. furnissii*; VAN: *V. anguillarum*; Con: consensus sequence). The RNase E cleavage sites are indicated.

While the 5′ end was generally considered to be the more important one of the two noncoding ends of bacterial mRNAs in the context of gene regulation, the growing list of functional 3′ end-derived sRNAs suggests that it clearly deserves more attention. These mRNA-derived fragments constitute an underappreciated layer of lateral gene control whereby a gene influences the expression of another after transcription has taken place. Importantly, many of the earlier described cases impressively illustrate that it is worth looking for sequence conservation at the 3′ end of genes beyond the typical Rho-independent terminator structures because such conserved sequences downstream of the stop codons might indicate the conserved seed sequences of such sRNAs (Fig. 9).

3′ UTR-derived sRNAs initially seemed to exhibit a narrow target spectrum and tended to impact their parental mRNAs or their functions—be it as a synergist as in the case of FarS (Huber *et al*. 2020) or, as shown for OppZ (Hoyos *et al*. 2020), as an antagonist. However, examples such as SroC, which sponges the GcvB sRNA (Miyakoshi *et al*. 2015a) and acts on other targets as well, have already blurred this simple view of 3′ UTR-derived sRNAs. Sponging as an alternative mode of action also appears more common than once thought, leading to questions as to whether sponging sRNAs should be considered a subclass of sRNAs rather than their own class (Denham 2020). Additionally, recent studies have predicted several sRNAs derived from 5′ UTRs or from within CDSs, even though it currently remains unknown whether these novel sRNAs have a similarly limited targetome to 3′ UTR-derived sRNAs or whether they act globally akin to intergenic sRNAs (Dar and Sorek 2018, Adams *et al*. 2021).

Regardless of the class of sRNA, the now established global techniques should be applied outside the commonly studied model organisms to yield a more comprehensive view of the abundance as well as the regulatory capacity of novel sRNAs. Herein lies yet another challenge, since most bacteria do not encode homologs of well-known RBPs or are not genetically tractable, rendering many approaches difficult if not impossible to apply. A point in case is the cancer-associated species *Fusobacterium nucleatum*. A recent RNA census of this and related fusobacterial species identified about two dozen sRNAs, of which four derived from 3′ UTRs (Ponath *et al*. 2021). Due to the lack of known RBPs and the limited genetic tools available for *F. nucleatum* and other bacteria, future studies analyzing their full sRNA repertoires will need to rely on the establishment of global methods independent of genetic manipulation or the presence of a global RBP. This will be important as the examples described earlier highlight the diverse regulatory roles of 3′ UTR-derived sRNAs in facilitating adaption to environmental changes or stresses. Thus, by studying this subset of sRNAs, we will further unravel and expand the complex regulatory networks crucial for model organisms and medically relevant pathogens alike.

## Acknowledgments

We thank Lars Barquist, Svetlana Ðurica-Mitić, Youssef El Mouali and Kai Papenfort for helpful comments on the manuscript and Dr Sandy Westermann (www.scigraphix.com) for help with preparing the figures. We are grateful to the Vogel Stiftung Dr Eckernkamp for supporting FP with a Dr Eckernkamp Fellowship.

## Funding

This work was funded by a DFG (Deutsche Forschungs Gesellschaft) Gottfried Wilhelm Leibniz Award to Jörg Vogel (DFG Vo875‐18).
